# The reliability of neurobehavioral tests in a thai adult population

**DOI:** 10.1590/1980-5764-DN-2021-0115

**Published:** 2022-07-29

**Authors:** Ajchamon Thammachai, Ratana Sapbamrer, Juthasiri Rohitrattana, Siam Tongprasert, Surat Hongsibsong, Kampanat Wangsan

**Affiliations:** 1 Chiang Mai University, Faculty of Medicine, Department of Community Medicine, Chiang Mai, Thailand.; 2 Chulalongkorn University, Center for Safety, Health and Environment, Bangkok, Thailand.; 3 Chiang Mai University, Faculty of Medicine, Department of Rehabilitation Medicine, Chiang Mai, Thailand.; 4 Chiang Mai University, Research Institute for Health Sciences, School of Health Sciences Research, Chiang Mai, Thailand.

**Keywords:** Mental Status and Dementia Tests, Neurobehavioral Manifestations, Reproducibility of Results, Memory, Short-Term, Testes de Estado Mental e Demência, Manifestações Neurocomportamentais, Reprodutibilidade dos Testes, Memória de Curto Prazo

## Abstract

Early detection of decline in neurobehavioral (NB) performance requires reliable methods of testing. Although NB tests have been shown to be consistent and reliable in Western countries, there has been limited research in Asian populations.

**Objective:**

The purpose of this study was to investigate the test-retest reliability of NB tests in a Thai adult population and examine the impact of demographic data on NB tests. The aspects of the tests chosen were memory, attention, hand-eye coordination, motor speed, and dexterity.

**Methods:**

The three NB tests used were digit span, Purdue Pegboard, and visual-motor integration. All three were administered to a population of 30 Thai adults.

**Results:**

The outcomes of all Pearson’s correlation coefficient tests (r) were positive and greater than 0.60, and subtest-retest reliability correlation coefficients ranged from 0.63 (p<0.001) to 0.81 (p<0.001). Interestingly, the outcomes of all of these tests were not affected by demographic data, with the exception of the Purdue Pegboard test, in which performance on the preferred hand and both hands assessment was weakly associated with age (β=-0.09, p<0.001 and β=-0.08, p<0.05, respectively).

**Conclusions:**

NB tests have adequate reliability and are useful for the evaluation of clinical memory, attention, hand-eye coordination, motor speed, and dexterity in Thai adults. These tests were not affected by demographic data. However, further studies to measure the validity of the digit span, Purdue Pegboard, and visual-motor integration tests are needed.

## RESUMO

A detecção precoce do declínio no desempenho neurocomportamental (NC) requer métodos confiáveis de teste. Embora os testes NC tenham se mostrado consistentes e confiáveis em países ocidentais, as pesquisas em populações asiáticas ainda são limitadas.

**Objetivo:**

O objetivo deste estudo foi investigar a confiabilidade de teste-reteste dos testes NC em uma população adulta tailandesa e o impacto dos dados demográficos nos testes NC. Os aspectos dos testes escolhidos foram memória, atenção, coordenação óculo-manual, velocidade motora e destreza.

**Métodos:**

Os três testes RC utilizados foram o *digit span*, o *Purdue Pegboard* e a integração visomotora. Todos os três foram usados em uma população de 30 adultos tailandeses.

**Resultados:**

Os resultados de todos os testes de coeficiente de correlação de Pearson (*r*) foram positivos e superiores a 0,60, e os coeficientes de correlação de confiabilidade subteste-reteste variaram de 0,63 (p<0,001) a 0,81 (p<0,001). Curiosamente, os resultados de todos esses testes não foram afetados pelos dados demográficos, com exceção do teste *Purdue Pegboard*, no qual o desempenho na mão preferida e a avaliação de ambas as mãos foi fracamente associado à idade (β=-0,09, p<0,001 e β=-0,08, p<0,05, respectivamente).

**Conclusão:**

Os testes NC apresentam confiabilidade adequada e são úteis para avaliação da memória clínica, atenção, coordenação óculo-manual, velocidade motora e destreza em adultos tailandeses. Esses testes não foram afetados por dados demográficos. No entanto, são necessários mais estudos para medir a validade dos testes de *digit span, Purdue Pegboard* e IVM.

## INTRODUCTION

The discipline of neurobehavioral (NB) toxicology has expanded to encompass approaches for the detection of subclinical abnormalities. Research in this field has focused on the use of traditional neuropsychological tests to identify atypical cases. NB test batteries have been commonly used to assess the impact of acute pesticide exposure on the NB in adult occupational populations^1^. One study discovered a relationship between occupational exposure and impairment in psychomotor speed, executive function, visuospatial ability, working memory, and visual memory^2^. Computerized performance evaluations have recently been introduced, and they appear to be applicable for carrying out standardized efficient field investigations. However, earlier studies have shown that due to the demand for alphabetic knowledge, computerized examinations may not be appropriate for the assessment of individuals with lower levels of education^3^.

In this study, the Purdue Pegboard, visual-motor integration (VMI), and digit span tests were selected, assigned to specific cognitive domains, and were used with people with lower education levels. The Purdue Pegboard is a low-cost, simple method for assessing fine motor skills, which has been shown to have strong test-retest reliability^4^. The Purdue Pegboard can be used to assess the ability of an applicant to perform an activity that requires hand dexterity and involves sensorimotor motor-related regions as well as the basal ganglia striatum^5^. In clinical and research settings, VMI is a widely used and standardized procedure. The VMI Beery-Buktenica Developmental Test is used to examine VMI, visual perception, and motor coordination impairment, which require cerebellar, brainstem, and frontal lobe function^6,7^. The digit span test is a memory and attention function test that requires the learning of digit sequences involving the right dorsolateral prefrontal cortex and superior frontal gyrus^8,9^. Attention and executive function have been linked to backward digit span. Forward digit span, in contrast, has been linked to short-term rote auditory memory^10^.

According to several authors in earlier investigations, NB tests are adequately consistent and reliable^11,12^. In Western nations, traditional NB tests and those administered using computers have been established and standardized; however, there has been little research into their reliability in Asian populations^13^. It is acknowledged that various factors associated specifically with Asian populations, for example, race and culture, could influence NB performance^14^. Furthermore, characteristics such as education, age, ethnicity, and cultural background could all influence the consistency of performance^15^. As a result, the detection methods developed for Western cultures need to be translated and modified to accommodate a new cultural context^12^. The modified digit span, Purdue Pegboard, and VMI were created specifically for Thai children who had been exposed to pesticides, but these tests have only been used with children^16,17^. Very few studies have investigated the potential impact of hazards on the cognitive development of Thai adults. To verify the potential future usefulness of NB tests in this population, this study aimed to investigate the test-retest reliability of NB tests, which included those assessing memory, attention, hand-eye coordination, motor speed, and dexterity. This study also examined the impact of demographic data on the testing.

To the best of our knowledge, this the first study of this type in a Thai community that examines the test-retest reliability for the digit span, Purdue Pegboard, and VMI. We also aimed to develop instructions for the administration of the tests facilitating their broader use in the prevention of cognitive decline in the Thai population.

## METHODS

### Participants

To determine the test-retest reliability of these NB tests, 30 participants between the ages of 25 and 65 years who were fluent in Thai and had no history of intellectual, mental, physical, or cognitive impairment participated in the tests. To reduce the risk of measurement error caused by transitory swings in anxiety, motivation, attention, and exhaustion, participants were instructed to have adequate sleep, avoid drug and alcohol use, and limit smoking on the days prior to the tests. The study was thoroughly explained to participants who then completed the consent form. The study was approved by the Ethics Committee of Chiang Mai University Faculty of Medicine’s Research Ethics in Humans (COM-2563-07707).

### NB tests

Three non-computerized NB tests were delivered by an examiner, namely, digit span, Purdue Pegboard, and VMI. These tests, which were originally part of the Behavioral Assessment and Research System (BARS), have been modified and enhanced for use with children aged 5 years and older^18,19^. It has been translated into other languages, including Thai, and was piloted by a research team before being used in previous study. A bilingual co-investigator translated all test stimuli and standardized instructions into Thai and then back-translated them^17^. All examiners were trained in administration by a psychologist (psychometric testing) and experienced investigators.

### Digit span test

The process for the digit span forward and backward tasks was modified in this version, and the number order was pseudo-randomized to avoid repetition. There are two sections to this task. The initial step is to digit span forward, which involves repeating numerals in the same order as they were received. At a rate of roughly one per second, the investigator pronounces a succession of digits. The list is then repeated by the participant in the same sequence. Following that, participants must reverse or backward order digits in the digit backward test.

In the digit span forward, the length of sequences gradually increases. The test begins with a two-number sequence and gradually increases to nine. Different sets of digit span forward tasks were employed in Trial 1 and Trial 2. Trial 1 is completed before Trial 2 to test the cognitive flexibility component. The digit span backward task is approached in the same way as the digit span forward task, with the exception that the longest list has eight items. The span scores are represented by five different values. The sum of the accurate digit span forward and backward responses from Trial 1 and Trial 2 is initially two values. In this study the maximum digit length achieved by each participant, specifically the longest sequence that they could correctly answer in both digit span forward and backward, was determined. Finally, the total subtest score was calculated by summing the results of both the forward and backward digit span tasks.

### Purdue Pegboard test

In the Purdue Pegboard (Lafayette Instrument Company, USA) test, the investigator followed the testing procedure as described in the Purdue Pegboard test user instructions for Model 32020A. There are three components to the test, namely, right hand, left hand, and both hands. Two vertical rows of 25 small holes run down the center of the exam board, with 4 cups across the top. Each of the 2 exterior cups has 25 pins. The processes used for administration and scoring the test were as follows: participants must use their dominant hand first, then their non-dominant hand to place as many pins as possible along each row within 30 s. This completes the right-hand and left-hand subtests. In the both hands subset, the test is bimanual and participants use both hands at the same time to place as many pins as possible down both rows in 30 s. The number of pairs of pins placed in 30 s determines the score for this subtest. For each subtest, the individual was instructed to carry out the test twice. The average number of pins placed in the allocated time was used to calculate the score for each of these subtests.

### VMI test

The Beery-Buktenica developmental test of visual-motor integration (VMI-6th) was the VMI tool used in this study^20^. In this paper-and-pencil test, participants must copy increasingly complicated designs. The full format test consists of 30 items that use geometric shape drawing to assess VMI. The Beery VMI was typically given in a single session and took 10–15 min to complete^21^. For each test score, a summary of raw scores and standardized scores was calculated, and the findings were presented and analyzed in terms of both raw and standardized scores.

### Procedures

The participants were evaluated twice at an interval of 2 weeks. All NB tests were carried out in a quiet setting, with just one adult being evaluated at a time. An investigator gave the participants instructions at each test station. A well-trained investigator monitored the practice tests to ensure that the participants understood the instructions. The investigator also offered encouragement in order to keep the participant’s attention on the examination.

To reduce inter-investigator variation in the test administration procedure, and also the impact of participant judgment on scoring, the same instructor evaluated and scored all NB tests. In both trials, participants were given similar fundamental ambient conditions, such as a comfortable room temperature, enough lighting, and a quiet setting.

### Statistical analysis

The statistical analysis was carried out using SPSS for Windows. Data pertinent to demographics were analyzed using frequency, mean, standard deviation, and range. All parameters were tested for normality using the Kolmogorov-Smirnov and Shapiro-Wilk tests. The mean values and standard deviation of each variable were calculated. Pearson product-moment correlations were calculated and utilized to investigate test-retest reliability. Recommendations regarding five reliability cutoff values made in a previous study were used as guidelines for this study^22^. These cutoff values were as follows: coefficients of 0.10 and below represent negligible correlation, coefficients between 0.10 and 0.39 represent a weak correlation, coefficients between 0.40 and 0.69 represent a moderate correlation, coefficients between 0.70 and 0.89 represent a strong correlation, and coefficients of 0.90 and above are considered very strong. Linear regression was used to analyze the effect of demographic data such as age, gender, education, income, and occupation. A p-value <0.05 was accepted as statistically significant.

## RESULTS

### Descriptive analysis

Table 1 shows the demographic information for all participants. The majority of the participants were female and married, with a mean age of 51.4±13.5 years. The majority of participants were Thai (90%) and had at least finished primary school as their level of education (66.7%). The three most common occupations among the participants were an employee (29.7%), housewife (24.3%), and farmer (18.9%), with monthly income ranging from 4,500 to 10,000 Baht.

**Table 1:** Demographic data of participants (n=30).

Parameters		n (%)	Mean±SD (min–max)
Gender	Male	6 (20.0)	
	Female	24 (80.0)	
Status	Single	3 (10.0)	
	Married	20 (66.7)	
	Divorced	7 (23.3)	
Race	Thai	27 (90.0)	
	Other	3 (10.0)	
Occupation	Farmer	7 (18.9)	
	Employee	11 (29.7)	
	Housewife	9 (24.3)	
	Merchant	3 (8.1)	
Health	Underlying disease	4 (13.3)	
	No underlying disease	26 (86.7)	
Income (baht)	<4,500	11 (16.7)	
	4,500–10,000	15 (50.0)	
	10,000–15,000	1 (3.3)	
	15,000–20,000	3 (10.0)	
Education	Primary education	20 (66.7)	
	Secondary education	9 (30.0)	
	Higher education	1 (3.3)	
Dominant hand	Right	27 (90.0)	
	Left	3 (10.0)	
Age			51.4±13.5 (25.0–65.0)
Weight (kg)			64.9±15.8 (44.0–104.0)
Height (m)			1.6±0.1 (1.5–1.7)
Body mass index			26.1±0.9 (18.7–35.6)
Systolic blood pressure (mmHg)			126.3±9.8 (107.0–140.0)
Diastolic blood pressure (mmHg)			75.8±8.6 (63.0–96.0)
Heart rate (bpm)			85.7±11.5 (66.0–108.0)

SD: standard deviation.

### Test-retest reliability

Table 2 shows the mean values, standard deviations, and correlation coefficients for the subtests of the test-retest reliability administration. Based on the criteria described by Schober et al.^22^, reliability estimates for each subtest ranged from moderate (0.40–0.69) to strong (0.70–0.89). All reliability correlation coefficients were positive and greater than zero. The test-retest reliability correlation coefficients of subtests were 0.66 (p<0.001) to 0.81 (p<0.001) for the digit span subtests. For the Purdue Pegboard, the test-retest reliability correlation coefficients of subtests were 0.73 (p=0.005) to 0.78 (p<0.001). Finally, the test-retest reliability correlation coefficients for the VMI raw score were 0.72 (p<0.001) and 0.63 (p<0.001) for VMI standard score.

**Table 2:** Test-retest reliability of digit span, Purdue Pegboard, and visual motor integration tests (n=30).

Studied tests	Subtest	1st	2nd	Correlation	
		Mean±SD	Mean±SD	r	p-value
Digit span	Correct score digits forward	10.00±2.46	10.20±2.16	0.81*	≤0.001
	Maximum digits forward	6.50±1.28	6.87±1.11	0.68*	≤0.001
	Correct score digits backward	3.63±1.29	3.60±1.47	0.66*	≤0.001
	Maximum digits backward	3.10±0.84	3.07±0.83	0.68*	≤0.001
	Total correct score	13.63±3.18	13.80±3.03	0.75*	≤0.001
Purdue Pegboard	Preferred hand	14.12±1.96	14.82±1.69	0.78*	≤0.001
	Non-preferred hand	13.68±1.61	13.82±1.79	0.76*	≤0.001
	Both hands	11.65±1.62	11.57±1.87	0.73*	0.005
Visual motor integration	Raw score	22.00±2.70	22.20±2.85	0.72*	≤0.001
	Standard score	74.90±14.65	74.87±14.96	0.63*	≤0.001

SD: standard deviation.

* p<0.001.

### Effect of demographic characteristics on NB tests

Age was negatively associated with performance on the preferred hand and both hands only in Purdue Pegboard (β±SE=-0.09±0.03 and -0.08±0.03, respectively). Education, gender, income, and occupation were not associated with the subtests of Purdue Pegboard. Performance on digit span and the VMI was not significantly affected by age, education, gender, income, or occupation. The results of these analyses are presented in Table 3.

**Table 3:** Effect of demographic data on digit span, Purdue Pegboard, and visual motor integration tests (n=30).

Studied tests	Subtest	β±SE (95%CI)				
		Age	Education	Gender	Income	Occupation
Digit span	Correct score digits forward	-0.01±0.04	0.88±1.08	1.48±1.12	0.09±0.54	0.55±0.46
		(-0.10, 0.08)	(-1.34, 3.10)	(-0.84, 3.79)	(-1.02, 1.19)	(-0.40, 1.51)
	Maximum digits forward	-0.01±0.02	0.29±0.55	0.68±0.57	0.09±0.27	0.31±0.24
		(-0.05, 0.04)	(-0.84, 1.42)	(-0.50, 1.86)	(-0.48, 0.65)	(-0.15, 0.83)
	Correct score digits backward	-0.40±0.03	-0.34±072	0.35±0.75	0.09±0.36	0.63±0.31
		(-0.10, 0.02)	(-1.82, 1.13)	(-1.19, 1.88)	(-0.64, 0.83)	(-0.01, 1.26)
	Maximum digits backward	-0.02±0.02	-0.08±0.40	0.28±0.42	0.13±0.20	0.33±0.17
		(-0.05, 0.02)	(-0.91, 0.75)	(-0.59, 1.14)	(-0.28, 0.54)	(-0.02, 0.69)
	Total correct score	-0.05±0.56	0.54±1.44	1.82±1.50	0.18±0.72	1.18±0.62
		(-0.17, 0.07)	(-2.43, 3.50)	(-1.26, 4.91)	(-1.30, 1.66)	(-0.09, 2.45)
Purdue Pegboard	Preferred hand	-0.09±0.03	-0.15±0.68	0.87±0.71	0.17±0.34	0.35±0.29
		(-0.14, -0.03)*	(-1.56, 1.25)	(-0.59, 2.34)	(-0.53, 0.87)	(-0.25, 0.96)
	Non-preferred hand	-0.09±0.03	-0.43±0.78	0.12±0.82	-0.13±0.39	0.34±0.34
		(-0.16, -0.03)	(-2.04, 1.19)	(-1.56, 1.81)	(-0.94, 0.67)	(-0.32, 1.04)
	Both hands	-0.08±0.03	-0.14±0.83	1.11±0.87	-0.05±0.41	0.52±0.36
		(-0.15, -0.01)+	(-1.85, 1.58)	(-0.68, 2.89)	(-0.91, 0.80)	(-0.22, 1.26)
Visual motor integration	Raw score	-0.09±0.05	0.21±1.28	-1.07±1.42	0.02±0.67	0.63±0.58
		(-1.19, 0.01)	(-2.85, 2.44)	(-3.99, 1.86)	(-1.37, 1.41)	(-0.58, 1.83)
	Standard score	-0.14±0.28	-0.06±7.61	-3.42±8.43	-0.02±3.99	2.77±3.47
		(-0.73, 0.44)	(-15.76, 15.64)	(-20.82, 13.99)	(-8.26, 6.28)	(-4.39, 9.23)

Values are presented as unstandardized β and standard error (SE), confidence interval (95%).

* p<0.001

+ p<0.05.

## DISCUSSION

This study aimed to investigate the test-retest reliability of NB tests in a population of Thai adults. The aspects of the tests focused on were memory, attention, hand-eye coordination, motor speed, and dexterity. This study also examined the impact of demographic characteristics on the tests. Our results showed a significant positive correlation in the test-retest of digit span, Purdue Pegboard, and VMI. There were moderate to strong correlation coefficients (r=0.63–0.81) in the digit span and VMI, while in the Purdue Pegboard, correlation coefficients were strong (r=0.73–0.78). Straub et al.^23^ suggested that acceptable reliability levels for a pilot study should be 0.60 or above.

The results of digit span tests were consistent with the study by Waters and Caplan^24^, who found that the reliability for backward digit span test is moderate (r=0.65). In addition, the reliability of the digit span was statistically significantly in the medium to high range for most of the Pearson’s correlation coefficients^24^. However, all of the correlations from these findings were higher than those of previous available studies. Rohitrattana et al.^17^ found that the reliability coefficients for maximum digits forward and backward in Thai children were 0.41 and 0.48, respectively. It is possible that children aged 6–8 years have lower levels of attention control than adults due to their susceptibility to auditory distraction^25,26^. Farahat et al.^27^ found that the reliability coefficients for forward and backward digit span in healthy population were 0.35 and 0.62, respectively. The low reliability coefficients might be due to the differences in measurement between the verbal and computerized digit span tests. Lower reliability in computerized digit span tests might be a result of a decline in visuospatial processes^28^.

With regard to the impact of demographic characteristics on the digit span, our findings suggested that demographic characteristics had no effects on the data. However, the results contradicted other previous studies^29–32^. Zimmermann et al.^29^ and Ostrosky-Solis and Lozano^30^ suggested that education and cultural context affected both forward and backward digit span tests. Farahat^31^ claims that participants with a higher degree of education had much higher digit span ability in both forward and backward spans. To add weight to this finding, Peña-Casanova et al.^33^ reported that age, education level, and language had an effect on the digit span. The probable reason that the demographic characteristics had no effects on the digit span in this population of Thai adults is that most participants in this study had finished primary school; therefore, there were more likely to have the same level of performance in the digit span test^34^. Another possibility is that the study population were culturally and linguistically homogeneous. Therefore, this study suggested that the digit span may be used to test memory and attention in the Thai adult population without impacts of demographic characteristics.

When considering the reliability for the VMI test, our findings found that the reliability was 0.63 for the standard scores and 0.72 for the raw scores. The standard score reliability of this study was higher than a prior study that found a test-retest reliability intraclass correlation coefficient (ICC) value of 0.58^35^. However, this result was similar to the correlation coefficients for totally correct VMI previously reported^17^. The raw scores from the VMI test in our study were shown to be reliable (r=0.72). Brown et al.^36^ reported a strong correlation (r=0.77) for the test-retest reliability for the Development Test of the VMI (DTVMI) and Bahk et al.^37^ found 0.79 correlation coefficients for VMI-6th. In our results, the correlation coefficients for both the VMI standard score and the raw score were lower than those found in previous studies. It is possible that the length of interval testing had an effect on pattern memory. Beery and Beery^38^ showed a test-retest reliability of 0.88 for 1-week interval testing, while our study investigated 2-week interval testing. This indicates that practice effects with shorter interval duration may enhance sensorimotor integration^39^.

Another possibility is that racial, cultural, or ethnic variance might affect the reliability correlation^38,40^. In Thailand, the Beery VMI test was utilized in a prior study to assess hand-eye coordination in children, but this study employed it in adults. Before using this test in a new country, it should be pilot tested to guarantee that it is reliable and valid for that culture^19^. Our results suggested that demographic characteristics had no effects on the Beery VMI test. Beery and Beery^38^ stated that the standardized scores were the cumulative frequency distributions of the raw scores created for each age group. Leading test experts and professional organizations, however, stated that it should be used with caution.

With regard to the Purdue Pegboard test, our results were consistent with the study by Rohitrattana et al.^17^, who found that the Pearson’s correlation reliability coefficients for assessing Thai children showed a strong correlation (r=0.71–0.72). However, all of these correlations from our finding were higher than those of previous studies. It is possible that the number of trials per subtest had an effect on the reliability. Buddenberg and Davis^41^ found that the correlation coefficients ranged from 0.37 to 0.82 for one-trial administrations over intervals of 1–2 weeks. Doyen and Carlier^42^ tested for three-trial administrations and found that the reliability ranged from 0.81 to 0.89. Our results, therefore, suggested that the reliability of subtests by two-trial administrations was greater and still acceptable when compared to previous studies.

Our results found that age had an effect on the Purdue Pegboard in the preferred hand and in both hands. These results agreed with the study by Rohitrattana et al.^17^, who found that higher scores of Purdue Pegboard correlated with higher age. These results were also consistent with a study by Brito and Santos-Morales^43^ who found that age had an effect on motor speed and dexterity performance, even in samples of children. Gur et al.^44^ also found that motor speed and dexterity were negatively associated with age. The decline in performance was related to frontotemporal function with age. Decline in dexterity was caused by slow movement and kinematic changes^45^.

These tests are applicable to a broader population because demographic characteristics have no effect on the tests. Importantly, this is the first study in Thai adult population and shows a high reliability for the use of the digit span, Purdue Pegboard, and VMI. They could be applied in investigations into potentially hazardous occupations, for example, pesticide-related jobs. However, there are some limitations. The lack of normative data was found for the digit span subtests and a standard score for the VMI test. As a result, we were unable to demonstrate concurrent validity in our study. Although these findings can be used to inform and enhance future studies into cognitive behavior by using the digit span, Purdue Pegboard, and VMI tests, the sample sizes are rather small. Therefore, larger sample sizes in future research are needed.

This study showed that NB tests, specifically digit span, Purdue Pegboard, and VMI, had moderate to strong reliability. All tests in our study can be applied to enable the clinical assessment of working memory, attention, hand-eye coordination, motor speed, and dexterity in the Thai adult population. Interestingly, demographic characteristics had no effects on all tests, with the exception of the Purdue Pegboard test. Therefore, further studies are needed to assess the validity of NB tests and investigate in large sample sizes.
